# Design of a Low-Cost Small-Size Fluxgate Sensor

**DOI:** 10.3390/s21196598

**Published:** 2021-10-02

**Authors:** Xiaoyu Shen, Yuntian Teng, Xingxing Hu

**Affiliations:** Institute of Geophysics, China Earthquake Administration, No. 5 Minzudaxue Nanlu, Haidian District, Beijing 100081, China; shenxy@cea-igp.ac.cn (X.S.); tengyt@cea-igp.ac.cn (Y.T.)

**Keywords:** compact magnetic sensor, fluxgate magnetometer, geomagnetic field measurement

## Abstract

Traditional fluxgate sensors used in geomagnetic field observations are large, costly, power-consuming and often limited in their use. Although the size of the micro-fluxgate sensors has been significantly reduced, their performance, including indicators such as accuracy and signal-to-noise, does not meet observational requirements. To address these problems, a new race-track type probe is designed based on a magnetic core made of a Co-based amorphous ribbon. The size of this single-component probe is only Φ10 mm × 30 mm. The signal processing circuit is also optimized. The whole size of the sensor integrated with probes and data acquisition module is Φ70 mm × 100 mm. Compared with traditional fluxgate and micro-fluxgate sensors, the designed sensor is compact and provides excellent performance equal to traditional fluxgate sensors with good linearity and RMS noise of less than 0.1 nT. From operational tests, the results are in good agreement with those from a standard fluxgate magnetometer. Being more suitable for modern dense deployment of geomagnetic observations, this small-size fluxgate sensor offers promising research applications at lower costs.

## 1. Introduction

A fluxgate sensor is a vector sensor that can be used to measure constant magnetic fields or low-frequency magnetic fields [1,2]. The sensor is based on the phenomenon of electromagnetic induction and modulates the measured magnetic field by the transformer effect. Compared with other types of magnetic sensors, fluxgate sensors have advantages such as high resolution (up to 0.1 nT), wide magnetic field measurement range and good stability [3,4,5,6,7]. 

At present, the main magnetic measuring instruments with resolution up to nT level are the fluxgate magnetometer, SQUID magnetometer, optical pump magnetometer and Overhauser magnetometer. Among them, the resolution of the optical pump magnetometer can reach 0.01 nT, but the optical system and gas chamber in its structure are large in size and are characterized by high power consumption; moreover, only the scalar measurement of the magnetic field can be performed. The Overhauser magnetometer has a resolution of 0.01 nT and the power consumption is only 0.5 W at a sampling rate of 5 seconds, but it can also only perform scalar measurements of the magnetic field. Only the SQUID magnetometer is more sensitive than the fluxgate sensor among the current vector sensors [8,9,10]. Nevertheless, the high demand for liquid helium or nitrogen (for high temperature SQUIDs) and the limited dynamic range make their use limited. Considering its advantages and disadvantages, the fluxgate sensor is still the most suitable vector sensor for magnetic field observations requiring resolutions of 0.1 nT and an absolute accuracy between 1 nT and 100 nT, as needed to monitor Earth’s magnetic field [11,12].

The current fluxgate sensors installed in fixed stations are often limited in their use because of their large size, high cost, high power consumption and the need for constant maintenance during use [13,14,15,16]. In addition, geomagnetic stations used for fluxgate-sensor observations occupy a large area and have other requirements concerning the surrounding electromagnetic environment. Moreover, all building materials need to be non-magnetic and specially customized; hence, these sensors are very expensive to fabricate. With the rapid social economic development, the construction of roads and other infrastructures brought about by urban expansion have further narrowed the possible sites for geomagnetic stations. Stations that once produced good-quality observation data have become outdated with the spread of transportation networks and dense distribution of transmission lines. These persistent problems make the study of small-size fluxgate sensors that can be buried all-important. Compared with traditional fixed station installation, buried installations have the advantages of low cost, flexible-site selection and dense deployment within a region. Nowadays, some advanced fluxgate magnetometers, such as the Mag-03 series of Bartington, can realize the buried placement of the probes. However, its accessories, such as the Mag-03 MC-MB cylindrical probe mounting bracket, Mag-03 DAM data acquisition module, or SCU1 signal conditioning unit, are still bulky and cannot meet the needs of buried observation of the whole fluxgate.

To achieve these goals of flexible siting, convenience in monitoring and intensive deployment, the traditional fluxgate sensor needs to be improved. Current developments in fluxgate sensors are directed toward miniaturization, low power consumption, low cost and improved accuracy over a large measurement range [17,18,19,20]. Three main types of micro-fluxgate magnetic sensor are available: (1) CMOS-based devices with flat coils, (2) sensors with thin-film or microfabricated solenoids and (3) PCB-based devices with solenoids made from tracks and vias [21,22]. However, these micro-fluxgate sensors fabricated through modern high technology are not suitable for applications directed towards monitoring Earth’s magnetic field, because their resolution is only a few nanoteslas and the RMS noise is several to a dozen nanoteslas in practical applications [23,24,25,26,27,28,29,30,31,32]. Nonetheless, their size is substantially reduced compared with that of conventional fluxgate sensors and their power consumption is considerably reduced.

Considering the limitations of traditional fluxgate sensors and the unsuitability of micro-fluxgate sensors for the needs of geomagnetic field observations, we propose a compact fluxgate sensor that does meet those needs. Different from the split design of the traditional fluxgate, this fluxgate integrates the probes and data acquisition module. As its core, a Co-based amorphous alloy cold drawn and annealed by vacuum-melt drawing equipment was selected. An improved miniaturized racetrack probe structure was designed. By optimizing the weak signal detection circuit, the original low noise, high resolution, high stability and other excellent performance indicators of the traditional fluxgate sensor were ensured along with a reduction in size and power consumption of the sensor.

## 2. Core Material and Probe Design

**Figure 1:**
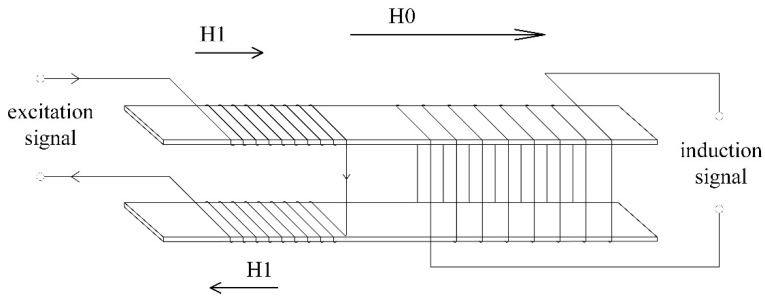
A dual-core fluxgate probe structure.

The fluxgate sensor exploits the non-linear relationship between the magnetic induction intensity and magnetic field intensity of a soft magnetic core under an alternating saturated magnetic field excitation to characterize the magnetic field as a voltage in measurements [33,34]. To reduce the interference signal generated by the transformer of the excitation signal, a dual-core fluxgate structure was selected (Figure 1). With this structure, the excitation coils are symmetrical in size and identical in electromagnetic parameter settings. When current is passed through the coils, the magnetic fields generated by them oppose. The magnetic fluxes generated by the excitation current through the common induction coils cancel each other, whereas the fluxes generated by the measured magnetic fields are superimposed on the induction coils.

**Figure 2:**
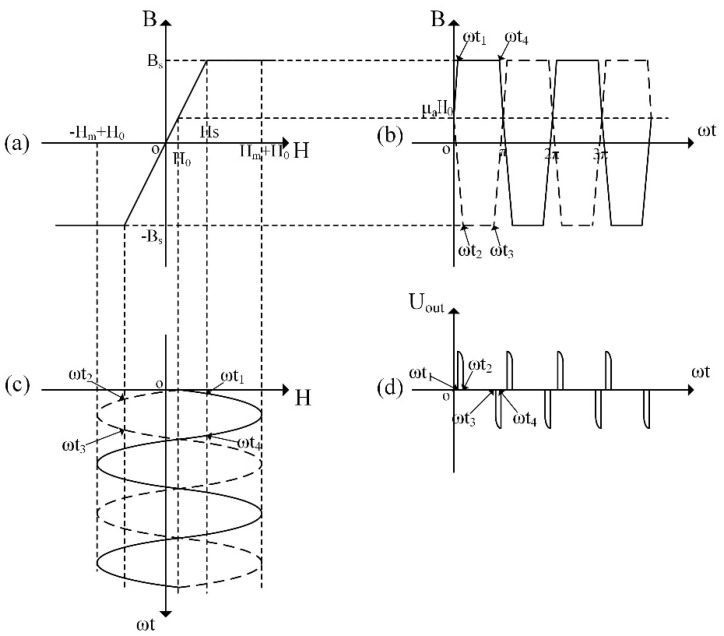
Schematic diagram of the working principle of the dual-core magnetic fluxgate sensor. (**a**) the simplified hysteresis line of the core (**b**) the core’s magnetic induction intensity (**c**) the core’s magnetic field intensity (**d**) the output voltage of the induction coil.

As shown in Figure 2a, a simplified trifold line is used to represent the hysteresis line of the core. When the magnetic field intensity is less than the saturation magnetic field intensity $H_{s}$ of the core, the magnetic permeability of the core is assumed constant at $\mu_{a}$. The excitation coils are excited by a standard sinusoidal current. Without considering the demagnetization, for example, of the core and eddy currents, a magnetic field with intensity $H_{m}\sin\omega t$ is generated inside the core. During operations, if the measured magnetic field intensity is $H_{0}$, the core’s magnetic induction intensity and its magnetic field intensity along with the output voltage of the induction coil are prescribed as illustrated in Figure 2b–d, respectively.

In accordance with Faraday’s law of electromagnetic induction, the output voltage of the induction coils in one period (0–π) is

(1) $$U_{out}=\left\{ \begin{array}{c} N_{2}\mu_{a}H_{m}S\omega \sin\omega t,\omega t_{1}\leq \omega t\leq \omega t_{2}\&\omega t_{3}\leq \omega t\leq \omega t_{4}\\ 0,otherwise \end{array}\right.,$$

where $N_{2}$ denotes the number of turns of the induction coils, S the cross-sectional area of the magnetic core, $\omega t_{1}=\arcsin\left(\frac{H_{s}-H_{0}}{H_{m}}\right)$, $\omega t_{2}=\arcsin\left(\frac{H_{s}+H_{0}}{H_{m}}\right)$, $\omega t_{3}=\pi -\omega t_{2}$ and $\omega t_{4}=\pi -\omega t_{1}$.

Performing a Fourier expansion on Equation (1) yields

(2) $$\begin{array}{cc} U_{out}= & \sum_{i=2}^{\infty }\frac{2N_{2}S\mu_{a}H_{m}\omega }{\pi }\{\frac{1}{i+1}\left[\cos\left(i+1\right)\omega t_{1}-\cos\left(i+1\right)\omega t_{2}\right]\sin i\\ & +\frac{1}{i-1}\left[\cos\left(i-1\right)\omega t_{1}-\cos\left(i-1\right)\omega t_{2}\right]\omega t\}\sin i\omega t. \end{array}$$

The amplitude of the second harmonic is the largest in the fluxgate induction output signal. From Equation (2), the output amplitude of the second harmonic is

(3) $$U_{2m}=\frac{8}{3\pi }N_{2}S\mu_{a}\omega \frac{1}{H_{m}^{2}}\left\{{\left[H_{m}^{2}-{\left(H_{s}^{2}-H_{0}^{2}\right)}^{2}\right]}^{\frac{3}{2}}-{\left[H_{m}^{2}-{\left(H_{s}^{2}+H_{0}^{2}\right)}^{2}\right]}^{\frac{3}{2}}\right\}.$$

When $H_{0}$ tends to 0, the sensitivity of the induced second harmonic is

(4) $$\begin{array}{c} G_{2}=\frac{dU_{2m}}{dH_{0}}=\frac{8}{\pi }N_{2}S\mu_{a}\omega \frac{1}{H_{m}^{2}}\{\left(H_{s}-H_{0}\right)\sqrt{H_{m}^{2}-{\left(H_{s}-H_{0}\right)}^{2}}\\ +\left(H_{s}+H_{0}\right)\sqrt{H_{m}^{2}-{\left(H_{s}+H_{0}\right)}^{2}}\}. \end{array}$$

When the measured magnetic field $H_{0}$ is small enough, requiring $\frac{dG_{2}}{dH_{m}}=0$ establishes the optimal excitation field amplitude of $H_{m}=\sqrt{2}H_{s}$ for the fluxgate sensor.

Taking into account the demagnetization of the core, the actual excitation magnetic field intensity H inside the core is

(5) $$H=\frac{1}{1+D(\mu_{r}-1)}\frac{N_{1}}{l}I_{m},$$

where D denotes the demagnetization coefficient, $\mu_{r}$ the relative permeability of the core, $N_{1}$ the number of turns of the excitation coils, l the length of the coil and $I_{m}$ the amplitude of the excitation current. Therefore, the optimal excitation current amplitude of the probe is

(6) $$I_{m}=\sqrt{2}\frac{l}{N_{1}}\left[1+D\left(\mu_{r}-1\right)\right]H_{s}.$$

The power consumption of the excitation current has a significant influence on the operation of the fluxgate sensor. To achieve low-power operation and reduce the magnitude of the excitation current, an analysis of the factors in Equations (4) and (6) shows that reducing $\mu_{r}$ reduces the required excitation current magnitude, but, at the same time, causes a reduction in the sensitivity of the probe. While $H_{s}$ is mainly determined by the material properties of the core, D and $l/N_{1}$ are mainly determined by the structure of the probe. Therefore, based on the traditional fluxgate sensor, we designed a miniaturized race-track type fluxgate probe with a core made of Co-based amorphous alloy ribbon.

Because the working principle of the fluxgate is based on the nonlinear magnetization properties of the core material, the core material needs to meet certain requirements, such as high relative permeability, low coercivity and low magnetostriction [35]. Therefore, when the external magnetic field changes slightly, the magnetic induction intensity in the core changes significantly and an electromotive force is generated in the induction coils. To reduce the power consumption of the sensor, the core material must have the lowest possible saturation magnetic field intensity. Therefore, permalloys and amorphous alloys with low saturation magnetic field intensity and high relative permeability are the best choices for the core.

The traditional fluxgate sensor has a core made of permalloys. Because of limitations in the processing technology, it is usually made into a ring, which makes the probe volume too large. At the same time, permalloys must undergo a rigorous heat treatment process at 1100 °C under stress, making the probe costly to manufacture. In contrast, amorphous alloy materials can, without heat treatment, acquire material properties close to permalloys, including good toughness, high mechanical strength and high permeability that adequately meets the requirements of cores for magnetic sensors [36,37,38,39,40,41]. The Fe-based amorphous alloys have high permeability characteristics, although the saturation magnetic field intensity is greater than that of Co-based amorphous alloys and the electroplated permalloy. Co-based amorphous alloys have good toughness, low saturation magnetic field intensity, high magnetic permeability and a near-zero magnetostriction constant, all of which are conducive in realizing low power consumption for the fluxgate sensor [42,43]. The core noise can be further decreased by annealing the Co-based amorphous alloy [29]. Magnetic permeability is also improved, making it more responsive to weak magnetic field signals.

**Figure 3:**
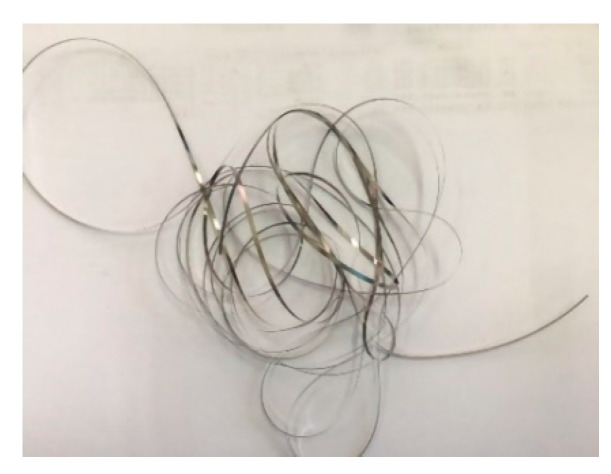
Co-based amorphous ribbon.

After comparison, the Co-based amorphous ribbon with a width of 1.6 mm (Figure 3) was selected as the core material, thereby improving the probe structure of the fluxgate sensor. The magnetic parameters of CACO-01 are shown in Table 1 below. Compared with the classic Metglas 2714A Co-based amorphous ribbon, it has a lower saturation magnetic field intensity and lower coercivity, making it more suitable for compact fluxgate probes.

**Figure 4:**
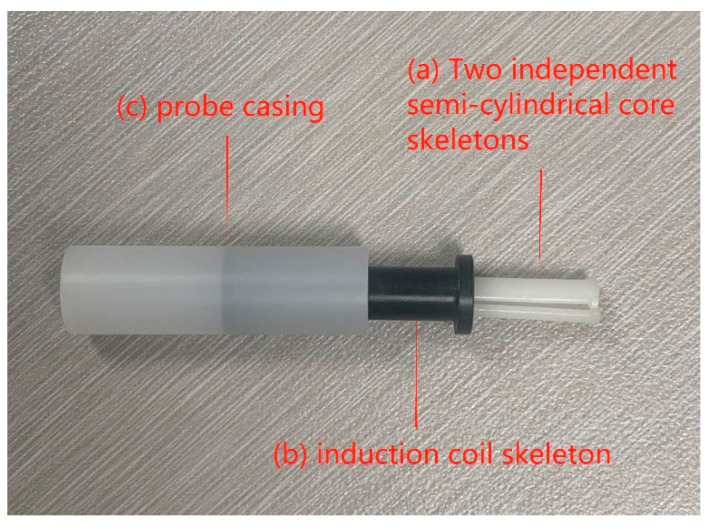
Photo of the probe structure. (a) Two independent semi-cylindrical core skeletons; (b) induction coil skeleton; (c) probe casing.

Despite its large size and high power consumption, the ring-type probe made of permalloy is highly sensitive. A conventional race-track type fluxgate probe does reduce the overall volume, compared with the ring-type probe. However, the narrow space of the short axis of the race-track type skeleton and the brittleness of the permalloy material after annealing mean that its fabrication can only be made by initially winding the magnetic core on the coil skeleton. Then, after high-temperature annealing, the wires are wound on the probe skeleton manually. The coils of this hand-wound race-track type fluxgate probe are not only difficult to wind, but incur a high manufacturing cost. Taking advantage of the good toughness characteristics of the Co-based amorphous alloy, we designed the main probe body based on the original race-track probe structure as two symmetrical and splittable semi-cylindrical skeleton structures (see Figure 4). The skeleton is made of epoxy resin, which has a low coefficient of thermal expansion and plexiglass material in the ratio of 5:1.

**Figure 5:**
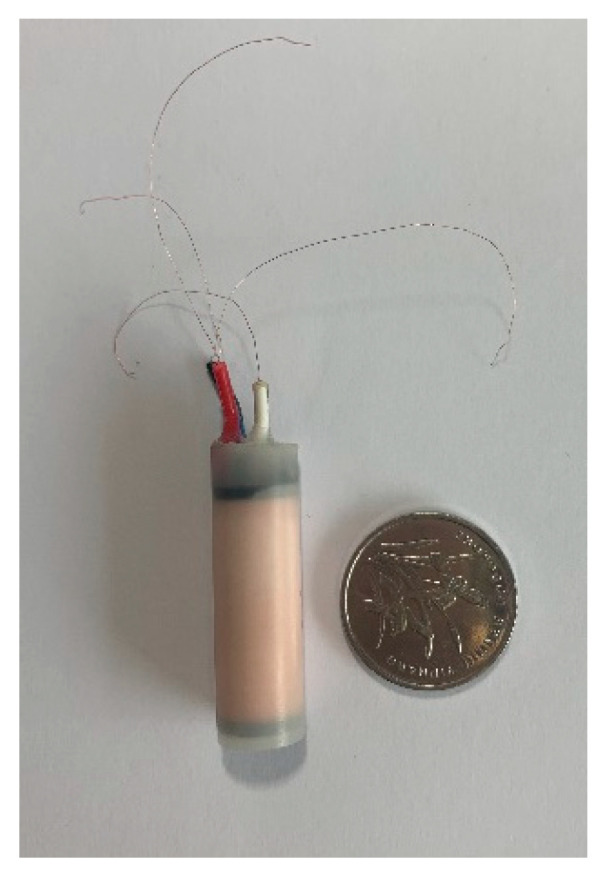
Photo of the single-component probe (a coin is used as a size reference).

Two independent semi-cylindrical core skeletons (Figure 4-a) enable the manufacturer to use a machine to wind the excitation coils, thereby significantly reducing labor costs. After winding is completed, the two skeletons are combined and fixed. A gap of 1.8 mm is left in the middle of the core skeletons that allows for the insertion of the Co-based amorphous ribbon. Amorphous alloy magnetic tapes reach periodic deep saturation as the high frequency excitation field changes. A multi-core design is adopted for the probe. This reduces considerably the required core cross-sectional area and excitation current compared with that using the traditional permalloy material, while maintaining the same probe sensitivity, thereby reducing the size and power consumption of the sensor. The induction coils (Figure 4-b) are also machine-wound. After winding, the core skeleton is inserted into the central cavity of the induction coil skeleton and encapsulated together into the externally protected probe casing (Figure 4-c). Compared with the size of the common ring-type probe of fluxgate sensors deployed in geomagnetic field stations, the size of the modified probe (Figure 5) is greatly reduced. The single-component probe is only Φ10 mm × 30 mm in size.

## 3. Concept of the Proposed Sensor and Realization

**Figure 6:**
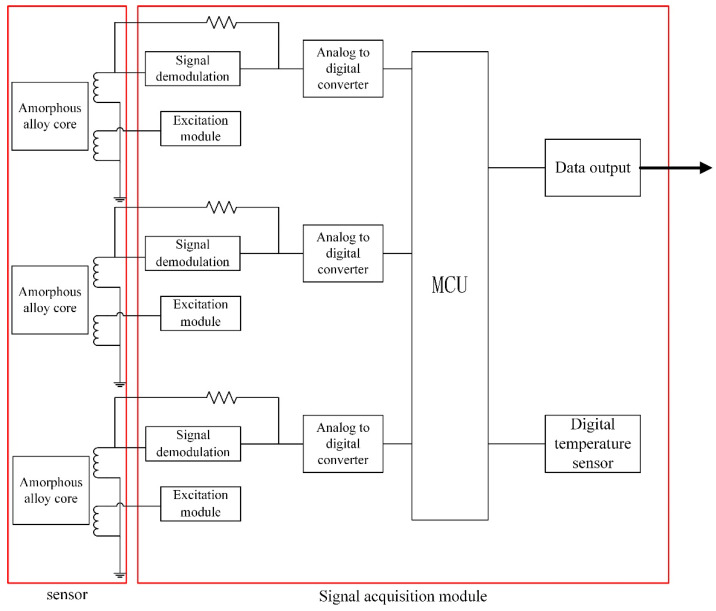
Simplified block diagram of the sensor processing circuitry.

The working principle of our small-size fluxgate sensor involves applying the second harmonic method to extract useful signals related to the measured magnetic field from the output voltage of the induction coil. In operation (Figure 6), the fluxgate sensor under the action of the excitation signal outputs a harmonic signal from the induction coil containing information about the change in the geomagnetic field. The alternating output signal is amplified using frequency selection, low-pass filtering, phase-sensitive detection, integration and other signal processing to form a quasi-DC voltage signal corresponding to the three components of the magnetic field, denoted H, Z and D.

In fluxgate sensor circuits, the excitation signal plays a decisive role. The parts concerning excitation and sensing are interlinked. When an error occurs in the excitation signal, the entire system undergoes a chain reaction that eventually leads to a wrong output from the probe sensing. Therefore, the excitation signal module of the fluxgate senor must have the following features: (1) high frequency stability, (2) high voltage amplitude stability, (3) high phase stability and (4) high waveform stability. The common means for generating excitation signals are crystal oscillators and timers of a microcontroller. The signals generated by crystal oscillators are not highly stable and require additional filtering for signal processing; they are therefore not suitable for small-size fluxgate sensors.

For this reason, a microcontroller was used to output the excitation signal, to have an adjustable frequency and a stable duty cycle and to ensure compactness. Both the excitation signal and the phase-sensitive demodulation reference signal in the signal processing circuit are generated by independent timers built into the microcontroller. While the signal is generated, a phase shift of the phase-sensitive demodulation reference signal is produced directly by the microcontroller, which ensures frequency stability and phase-shift accuracy. Once the excitation system is initialized, the control and intervention of the microcontroller is no longer required, thereby reducing the power consumption of the fluxgate sensor. To ensure this small-size fluxgate sensor still has high sensitivity, low noise and low power consumption, the excitation signal generated is a high-frequency triangular waveform.

## 4. Experiments and Results

**Figure 7:**
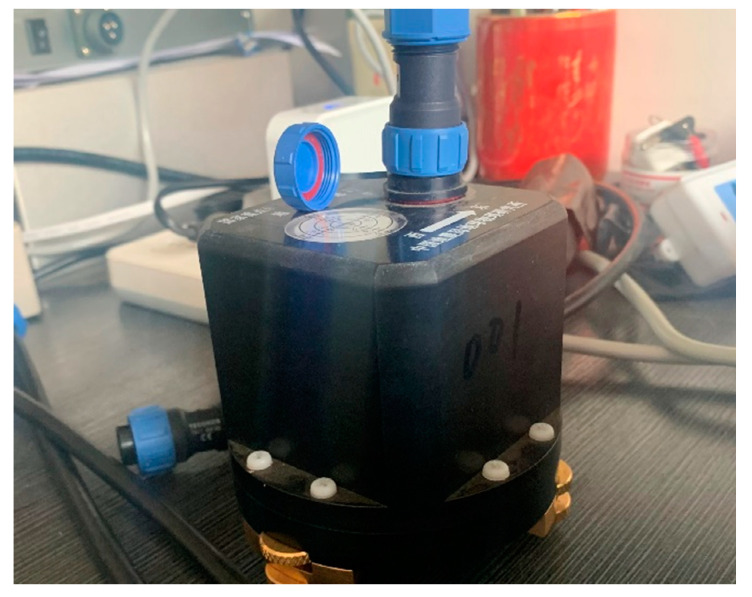
Photo of the fluxgate sensor.

**Figure 8:**
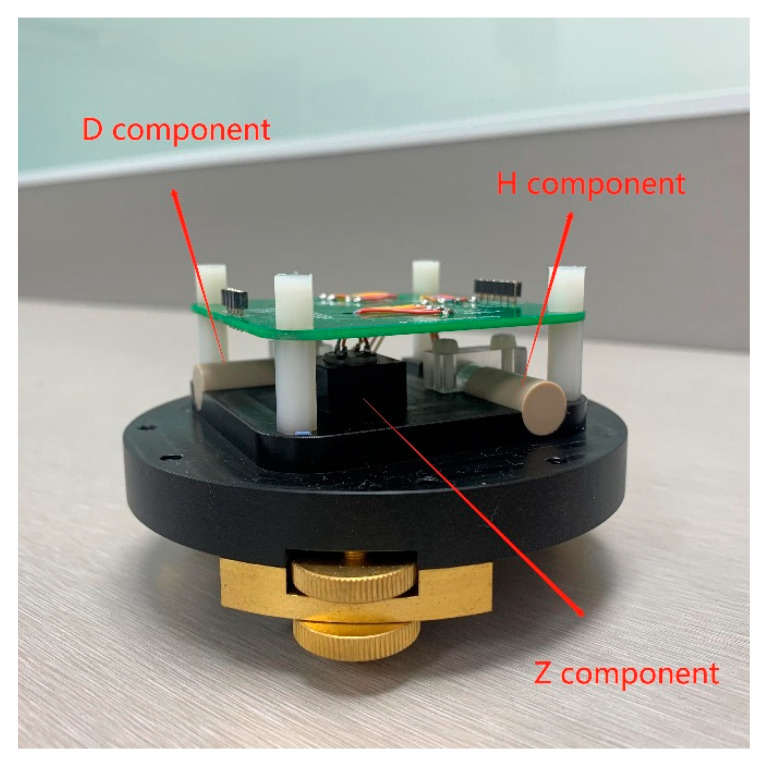
Location of the three probes.

With the designed small-size fluxgate sensor (Figure 7), the size of the whole device is only Φ70 mm × 100 mm. The location of the three probes in the device is shown in Figure 8. The H component probe and the D component probe are placed orthogonally in the horizontal plane and the Z component probe is placed on the vertical orthogonal plane.

**Figure 9:**
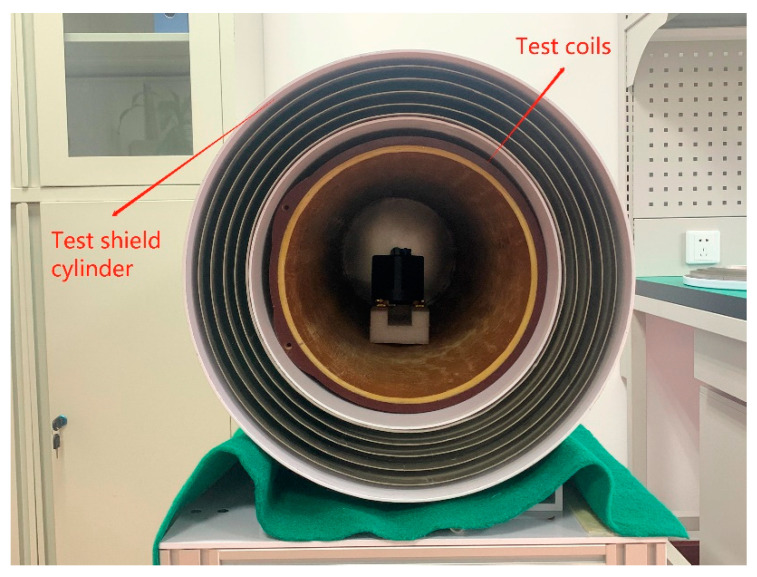
Test shield cylinder for determining the magnetic sensor performance.

In constructing a test platform for the small-size fluxgate sensor, we chose a magnetic-field-shielding cylinder (Figure 9) with a shielding space of more than 30 cm and a length of not less than 2 m, a permalloy shielding layer of not less than 6-layer thick, a loadable test coil, a high-precision current source and a high-precision signal generator. The fluxgate sensor was placed at the center of the shielding cylinder. The sensor and component probe to be measured were aligned parallel or coincident with the axis of the shielding cylinder.

### 4.1. Linearity Testing

**Figure 10:**
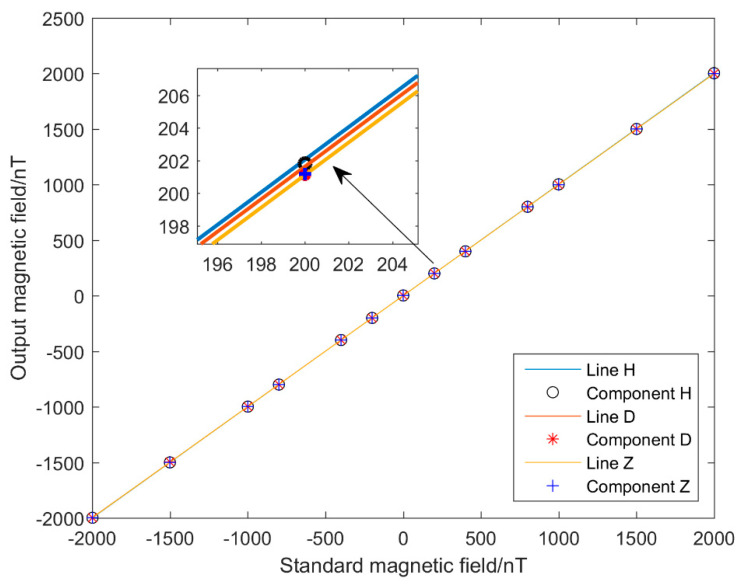
Linearity test results.

The current in the test coil of the magnetic shielding cylinder was varied to generate standard magnetic fields of different strengths. The output values of the fluxgate magnetometer were recorded for the different magnetic field strengths (see Table 2). The test results (Figure 10) show that the linearity of our fluxgate sensor was better than 4‰ along all three axes; with this excellent linearity, a good performance similar to the traditional fluxgate sensor was attained.

### 4.2. Noise Testing

**Figure 11:**
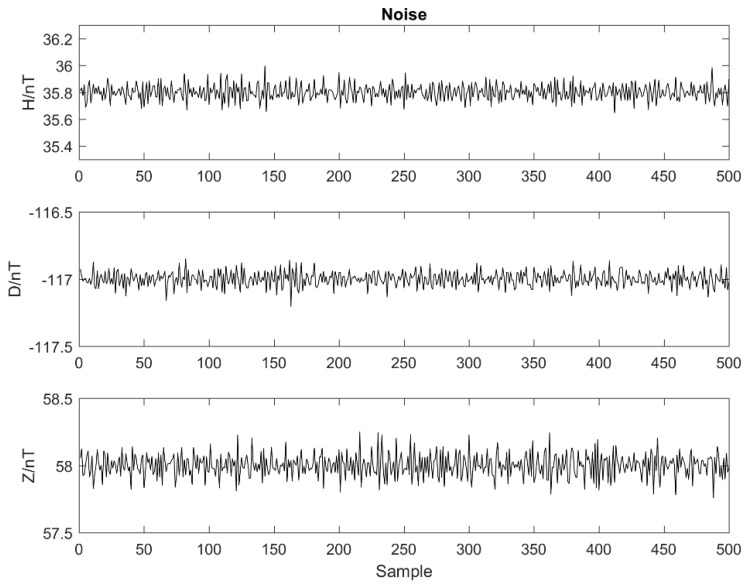
Three-component data waveform from the prototype fluxgate sensor in the shielding cylinder.

For noise tests, there was no applied magnetic field in any direction inside the shielded cylinder. The output values of the three components of the fluxgate sensor were recorded over a set period of time. Five hundred continuous samples of data were selected after readings were stabilized (Figure 11). The RMS values of noise, $Noise=\sqrt{\frac{1}{N-1}\sum_{i=1}^{N}{\left(B_{i}-\bar{B}\right)}^{2}}$, where N denotes the number of samples, $B_{i}$ the sampled value and $\bar{B}$ the average of the sampled values, were calculated and are here listed in Table 3. The maximum value among the three components of noise is 0.087 nT, that is, the designed fluxgate sensor has an RMS noise value below 0.1 nT. Compared with the noise level of traditional fluxgate devices used at geomagnetic field stations, such as the GM4 fluxgate magnetometer used by the Chinese geomagnetic network, the noise level of our sensor is comparable and meets the needs for geomagnetic field observations. Compared with a micro-fluxgate sensor, our sensor has significantly less noise.

### 4.3. Instrument Comparison Test

**Figure 12:**
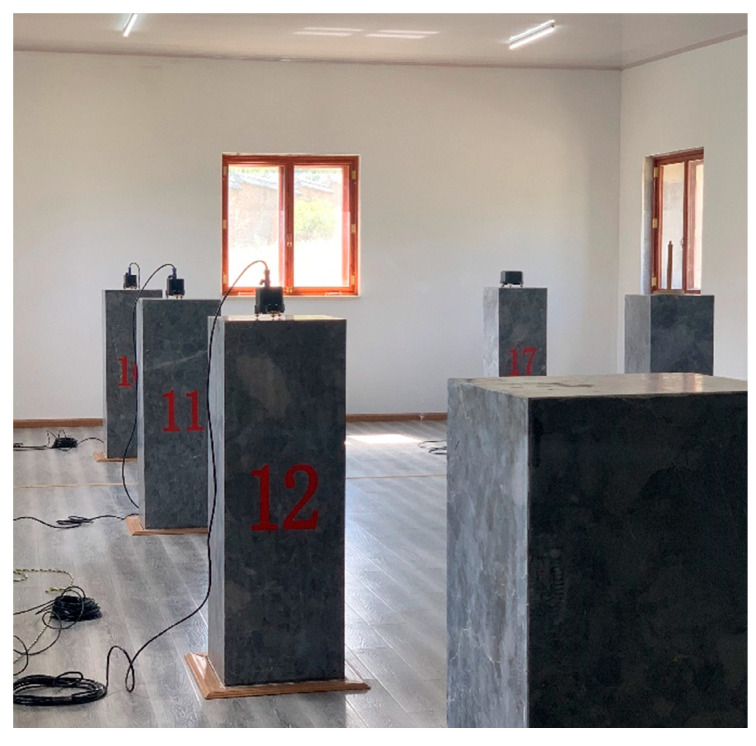
Photo of the observation experiment used for the fluxgate senor comparison.

A simultaneous experiment comparing our small-size fluxgate sensor with a standard fluxgate sensor of a geomagnetic reference station was performed (Figure 12). The type of the reference standard instrument is a GM4-XL fluxgate magnetometer.

**Figure 13:**
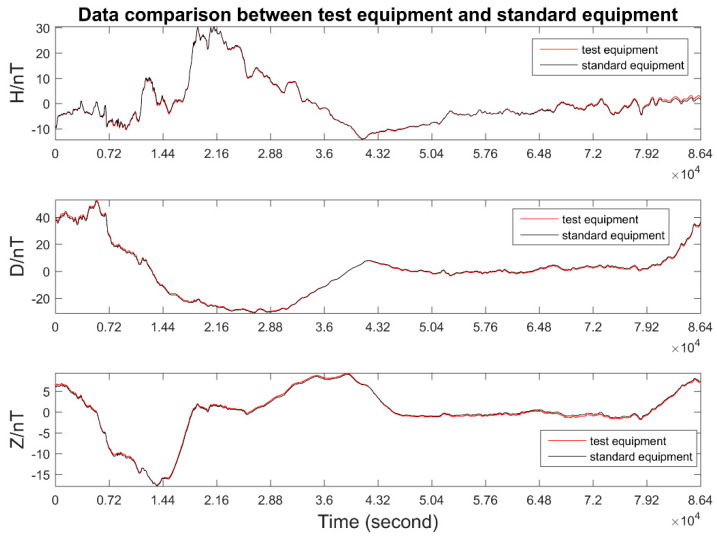
Daily variational curves of the test device and standard device.

The performance of the fluxgate sensor was further examined from a correlation analysis of the daily geomagnetic field variation recorded by the standard instrument and designed instrument. After a period of observations, the data for the three components of the geomagnetic field measured by the two instruments in the same working environment were in good agreement. The data taken over one day of recordings were plotted for a visual comparison (Figure 13).

Through data processing and analysis, the designed fluxgate sensor behaved highly consistently, compared with the standard instrument. The correlation coefficients obtained from the daily recordings of the three components of the geomagnetic field were all above 0.99. That is, there is good consistency and coincidence between the data sets of the two instruments. The designed fluxgate instrument truly reflects the variation in Earth’s magnetic field and meets the criterion needed for daily monitoring.

## 5. Conclusions

With a Co-based amorphous alloy ribbon as the magnetic core material in the fluxgate sensor, we designed a modified miniaturized race-track fluxgate probe based on the traditional race-track probe structure. The size of the single-component probe is only Φ10 mm × 30 mm. The sensor circuit was designed to employ the second harmonic method. The excitation signal of the sensor and the phase-sensitive demodulation reference signal were generated using the built-in timer of the microcontroller. Phase shifts were achieved directly by the microcontroller while the signal was generated, ensuring the stability of the frequency and accuracy of phase shifts. With experimental measurements, the fluxgate sensor showed good linearity and the RMS noise was less than 0.1 nT for each of the three field components. The agreement between the designed fluxgate sensor and the standard fluxgate sensor of the station is good and ensures the operational viability of the designed device. The proposed fluxgate sensor is compact and its power consumption is low and attains good linearity, low noise and excellent performance over a wide measurement range, all of which is needed for daily geomagnetic field monitoring. The comparison between the proposed fluxgate sensor and other sensors is shown in Table 4.

The small-size fluxgate sensor proposed makes up for the shortcomings in existing micro-fluxgate sensors, the performance of which does not meet the needs for field observations. Problems of large volume and power consumption of traditional fluxgate instruments were also solved. The designed sensor is more suitable for modern dense deployment of geomagnetic field stations and is conducive in solving the deteriorating quality in data from fixed geomagnetic stations through buried installations operating with magnetic fluxgate sensors. The application prospects of the fluxgate sensor are very broad.

## Figures and Tables

**Table 1:** The magnetic parameters of CACO-01 and Metglas 2714A.

Type	Composition	Saturation Magnetic Field Intensity/T	Coercivity /Am^−1^	Maximum Permeability
CACO-01	Co-Fe-Mo-Si-B	0.55	0.13	1,000,000
Metglas 2714A	Co-Fe-Ni-Si-B	0.57	0.4	1,000,000

**Table 2:** Linearity test results. All magnetic field values are given in nanoteslas.

Standard Magnetic Field	Component H	Linearity/‰	Component D	Linearity/‰	Component Z	Linearity/‰
2000	2003.99	1.15	2000.53	0.62	2000.53	0.23
1500	1503.37	1.12	1500.82	0.63	1500.74	0.17
1000	1002.9	1.21	1001.13	0.63	1000.91	0.08
800	802.39	0.88	800.83	1.16	800.73	0.33
400	401.87	0.45	401.36	1	401.01	0.05
200	201.83	0.7	201.12	3.2	201.17	0.9
0	1.69	/	1.76	/	0.99	/
−200	−198.56	1.25	−197.79	2.25	−198.887	0.615
−400	−398.55	0.6	−397.4	2.1	−398.67	0.85
−800	−798.7	0.49	−796.91	1.67	−798.37	0.8
−1000	−999.04	0.73	−997.24	1	−998.59	0.42
−1500	−1499.27	0.64	−1496.64	1.07	−1498.19	0.55
−2000	−1999.38	0.54	−1996.31	0.97	−1997.91	0.55

**Table 3:** Noise test results.

	Component H	Component D	Component Z
RMS noise (nT)	0.065	0.059	0.087

**Table 4:** Comparison between the proposed fluxgate sensor and other sensors.

Type	Operation Range	Size	Power Consumption	Linearity	RMS Noise
The proposed sensor	±70,000 nT	Φ70 mm × 100 mm ^1^	<2 W	<4‰	<0.1 nT
GM4	±62,500 nT	Φ180 mm × 100 mm ^2^	<4 W	<5‰	<0.1 nT
Mag-03	±70,000 nT	Φ25 mm × 202 mm ^2^	<3 W	<5‰	<0.1 nT
The sensor in [27]	±50,000 nT	5.5 mm × 5.8 mm ^2^	33.75 mW	/	23 nT
The sensor in [31]	0~100,000 nT	6.74 mm × 9 mm ^2^	20.35 mW	<4%	2.2 nT

^1^ The whole size of the device. ^2^ The size of the probe.

## Data Availability

Not applicable.
